# Articulating ethical principles guiding Target Malaria's engagement strategy

**DOI:** 10.1186/s12936-022-04062-4

**Published:** 2022-02-05

**Authors:** Aaron J. Roberts, Delphine Thizy

**Affiliations:** 1 Institute On Ethics and Policy for Innovation, McMaster University, Hamilton, Canada; 2 Imperial College, London, UK

**Keywords:** Ethics, Stakeholder engagement, Gene drive, Vector control, Malaria, Responsible research, Co-development

## Abstract

Progress in gene drive research has engendered a lively discussion about community engagement and the ethical standards the work hinges on. While there is broad agreement regarding ethical principles and established best practices for conducting clinical public health research, projects developing area-wide vector control technologies and initiating ambitious engagement strategies raise specific questions: who to engage, when to engage, and how? When responding to these fundamental questions, with few best practices available for guidance, projects need to reflect on and articulate the ethical principles that motivate and justify their approach. Target Malaria is a not-for-profit research consortium that aims to develop and share malaria control and elimination technology. The consortium is currently investigating the potential of a genetic technique called gene drive to control populations of malaria vectoring mosquito species *Anopheles gambiae*. Due to the potentially broad geographical, environmental impact of gene drive technology, Target Malaria has committed to a robust form of tailored engagement with the local communities in Burkina Faso, Mali, and Uganda, where research activities are currently taking place. This paper presents the principles guiding Target Malaria’s engagement strategy. Herein the authors (i) articulate the principles; (ii) explain the rationale for selecting them; (iii) share early lessons about the application of the principles. Since gene drive technology is an emerging technology, with few best practices available for guidance, the authors hope by sharing these lessons, to add to the growing literature regarding engagement strategies and practices for area-wide vector control, and more specifically, for gene drive research.

## Background

In 2021, the World Health Organization (WHO) published the 2nd edition of its guidance for testing genetically modified mosquitoes [1]. A major portion of that report was dedicated to ethical considerations, stressing and the importance of responsible community engagement. Ongoing discussions about genetically modified mosquitoes, including gene drive, tend to focus on ethical aspects of the research [2]. When considering different engagement strategies in the field of gene drive research, it is important to design strategies that are both ethical and effective. At least one other gene drive research programme with malaria elimination goals, the University of California Irvine Malaria Initiative, has published reflections on the formulation of their engagement methodology [3]. By describing their engagement model and its underlying principles, they took an important step towards transparency. This paper aims to do the same by articulating the ethical principles guiding the design and implementation of Target Malaria’s engagement strategy.

From the beginning Target Malaria, one of the earliest and most advanced gene drive research projects, initiated a process of formally articulating the project’s core values. These values remain in place today:

- Excellence,
- Co-development,
- Being evidence-driven, and
- Openness and accountability [4]

In further commitments, Target Malaria’s engagement strategy has had to align with these values in addition to other ethical principles. For instance, the value of excellence implies aligning with best practices and guidance available in the evolving literature concerning engagement practices for gene drive research.

In what follows, the paper articulates the principles which continue to guide Target Malaria’s evolving engagement strategy. The authors also explain the rationale for selecting these principles, which was informed by ethical values, including core project values, emerging guidance and developing best practices in the field. Due to the relative novelty of gene drive technology, the authors aim to contribute to the development of still evolving engagement and best practices in this field.

## Case presentation: Target Malaria and gene drive research

Target Malaria is a not-for-profit research consortium that aims to develop and share malaria control and elimination technology. It is currently researching the potential of a biotechnological phenomenon called gene drive to control populations of the mosquito species *Anopheles gambiae*, one of the main vectors of malaria. Gene drive is a term describing the preferential inheritance of a genetic trait in the offspring of a sexually reproducing organism. With the new technique, particular traits can be inherited by close to 100% of the offspring instead of the classical Mendelian inheritance rate, which is approximately 50% [5, 6]. This way, in a sexually reproducing population, traits can spread rapidly, even if the modification does not provide a fitness advantage to individuals who carry it [6]. Gene drive mechanisms are not a novel phenomenon; they occur in nature [5, 7]. In recent years, the original idea of utilizing gene drive to develop a new vector control tool was shown to be possible in the laboratory [8–10]. Given this progress, the Target Malaria strategy to use gene drive to reduce the population of malaria-transmitting mosquitoes below the threshold necessary for effective malaria transmission, interrupting malaria's local transmission in conjunction with other control methods [11], has become a realistic possibility.

Consistent with WHO guidance [1], Target Malaria follows a phased approach, with gene drive mosquitoes representing the ultimate phase: the emergence of a self-sustaining strain able to spread the modification to the target population. The initial phases involve using non-gene drive strains of genetically modified mosquitoes [12], which allow for iterative development, integrating learning from previous modified strains to the next phase. This modus operandi also leaves room for piloting the engagement strategy, developed in an early implementation stage according to principles presented here. This is followed by iterative refinement of the engagement strategy along with the changing context of the different research phases.

The nature of gene drive technology poses serious challenges to designing effective community engagement. This is due to the limited availability of past examples of quality engagement practices regarding the application of public health technologies in similar area-wide contexts. Because synthetic gene drives have not been evaluated in the wild and require a methodical stepwise testing pathway [12], communities need to be engaged about overall project goals and methods, as well as the specifics of each research stage. To complicate matters further, each research stage likely involves a different subset within the relevant community. Guidance documents about responsible engagement are still being developed [13, 14]. Target Malaria aims to continue developing its engagement strategy guided by the principles detailed in this article.

The importance of community engagement for a genetic approach to vector control has long been recognized [13–25]. It has been a focal point for Target Malaria since the project’s inception, even before its formal establishment as ‘Target Malaria’. There are good reasons for designing and enacting a robust engagement strategy around a research project aimed at area-wide vector control. These can be legal, financial, operational, or reputational in nature. However, the authors argue the most important is ethical [25]. This paper articulates Target Malaria's principles for guiding its engagement strategy and explains the rationale for selecting them, grounded in principles and values.

To date, papers and guidelines analysing ethical obligations inherent to engagement practices [1, 18, 23] have primarily focused on the question of consent to the release of genetically modified mosquitoes [13, 15, 18, 20, 26, 27]. They also suggested that engagement should include a greater degree of partnership than previously seen between researchers and communities living in the research area [3, 14, 18, 23, 24]. When faced with difficult decisions, well-articulated principles can guide a project’s strategy design. Still, very little has been written about how and why a given project selects the principles they will follow. This paper explains the rationale behind the four fundamental principles Target Malaria uses to guide its decisions around engagement. These principles are:

1. Prioritize engagement with the most ethically relevant groups
2. Conduct engagement in the spirit and form of co-development
3. Engagement activities should be conducted by representatives of the research project
4. Begin engagement early, engage continuously, and iterate often

The paper starts by briefly examining how Target Malaria’s project values align with and drive the implementation of a robust programme of principled engagement in the first place.

## How Target Malaria’s project values motivate commitment to principled engagement

Each of Target Malaria’s project values

- Excellence,
- Co-development,
- Being evidence-driven,
- Openness and accountability

are mutually reinforcing, orienting the project towards enacting robust community engagement. For instance, the pursuit of excellence requires for the project to achieve the highest standards of responsible research and best practices. Because a genetic approach to vector control is a fairly new field of research, with yet unestablished best practices, projects engaged in this research have a responsibility to work collaboratively with others to develop them. Excellence and being evidence-driven implies and requires rigorous research, both in the laboratory and in the communities and ecosystems where the research is conducted, to benefit the people the research aims to help. In Target Malaria's case, it is these same communities that bear most of the risk of the research.

From the start, Target Malaria has been committed to the values of co-development, openness, and accountability. The project prioritized these values because they are underwritten by a core tenet of ethical research: respect for persons [28]. Engaging affected parties openly and accountably with the aim of being granted consent—or in the case of a community-wide intervention like gene drive, community agreement [29]—reflects this respect for persons. This normative process is a prerequisite for any research activity that potentially impacts human beings. For this reason, the project’s values compel it to engage in this manner whether or not external requirements or regulations demand it. This is important since external requirements for engagement for gene drive research are in many cases still under development.

Commitment to the values of openness and accountability require that Target Malaria engages transparently with communities, stakeholders, and the public about its work. Being accountable implies a further commitment to the value of justice, both substantively and procedurally. This demands that the project avoid and/or manage a potential adverse impact on communities and the environment where they live. It also explores how benefits for communities and other stakeholders can be maximized by investing in local capacity building. The project should also give relevant communities the opportunity to voice their concerns and share with the project first-hand knowledge of their own needs and values and insight into their interaction with and dependence on the local environment. Doing so can help inform the project of a broader scope of relevant risks and related protection goals than could have been identified otherwise, further minimizing potential harm [24].

The value of co-development requires that communities are empowered with an informed and impactful voice in decisions that will affect them and the design of the processes by which those decisions are made. Because openness and accountability are analogues for honesty and trust [4], the engagement strategy needs to foster and build trust. The only way to do this is through numerous interactions over an extended period and consistent demonstration of honesty, reliability, accountability, and good-faith behaviour.

Although the above does not provide an exhaustive analysis, Target Malaria’s decision to make its project values explicit provides the ethical mandate for answering further questions regarding designing an ethical engagement strategy. These values subsequently informed the selection and articulation of principles to guide the design of an engagement strategy for Target Malaria’s gene drive research project. The remainder of this Commentary explains in greater detail the rationale behind the selection of each of Target Malaria’s engagement principles.

**Fig. 1:**
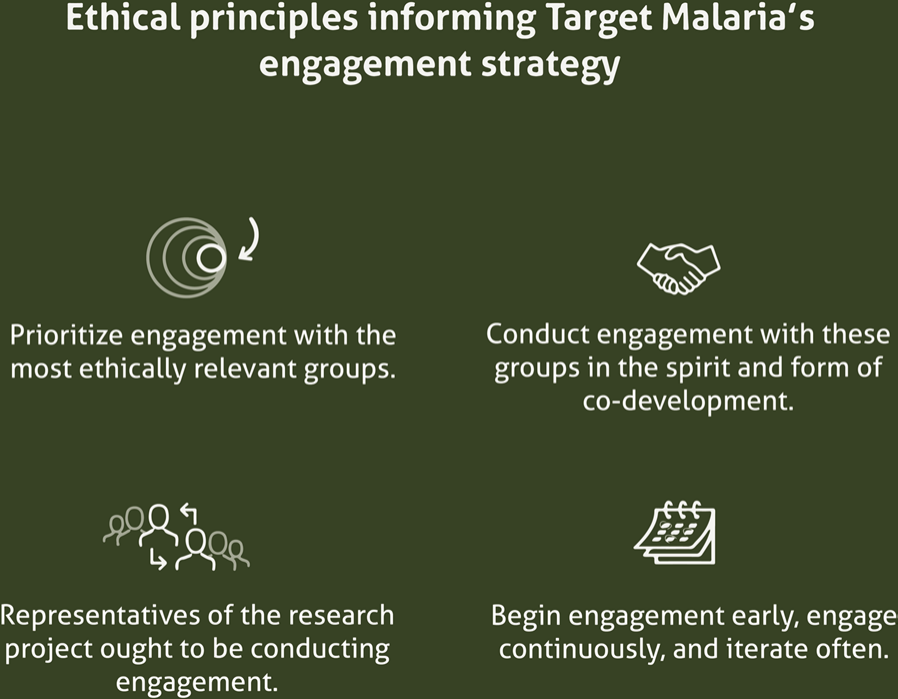
Guiding Principles for Ethical Engagement

For examples of how these principles (Fig. 1) inform Target Malaria's community engagement protocols and practices, please see “Small-scale release of non-gene drive mosquitoes in Burkina Faso: from engagement implementation to assessment, a learning journey” by Lea Pare Toe et al. [30].

### Prioritize engagement with the most ethically relevant groups

In reference to its recommendations around engagement practices, the 2016 report *Gene Drives on the Horizon*, published by the National Academies of Sciences, Engineering, and Medicine (henceforth, the NASEM Report), distinguishes between communities, stakeholders, and publics [23]. Each of these groups has some stake in the outcome of a gene drive project, and as such, all deserve to be engaged about the project in a manner appropriate to their context and needs. Nevertheless, the consequences of the research, whether harm or benefit, resulting from a decision to proceed with the research or not, will be borne to differing degrees by each of these groups. They will be borne most directly and heavily by the relevant communities. To engage each group, communities, stakeholders, and public in the same way or with the same prioritisation of time, resources, consideration, and importance would be inappropriate. Instead, in alignment with the project values of accountability and basing decisions on evidence, each group should be engaged with priorities proportional to their ethical relevance to the processes and results of the project. The degree of ethical relevance of a group is to be assessed via a formal impact analysis; how likely and significantly will they be affected as a consequence of the project’s activities, whether positively or negatively.

Of the three groups, communities, as defined by the NASEM report definition, have the greatest and most direct stake in the processes and outcomes of project activities. This greater stake grants them greater ethical relevance. Identification of a community’s ethical relevance necessitates a systematic review of how the project (both with the mosquito strain itself and proposed activities such as monitoring) will potentially impact the community socially, economically with regards to public health and their access to and use of ecosystem services [31]. It is only reasonable that more significant consideration should be offered to those most likely to be directly and consequentially impacted by the research process and results. This greater consideration should be demonstrated by prioritising and empowering the communities identified as most relevant to have meaningful input—throughout the project cycle—regarding the research project’s goals and the design of its processes [14, 32, 33].

The set of communities identifiable as the most ethically relevant will evolve throughout the project in relation to the proposed activities and the mosquito strain used (based on their potential dispersal and persistence), and the type of entomological activities. For example, the potential impact on communities' daily lives is different if the project collects mosquitoes using an insecticide spray and catch technique (which requires entering people’s homes) versus using the swarming capture technique (which takes place outside in the village common area) [34]. Several studies inform the project’s identification and analysis of community relevance, some directly carried out by the project (such as modelling studies), others by external experts or via considerations found in the growing engagement literature. Ultimately, the identification of ethically relevant communities is reviewed by the institutional ethics committee that, in the end, must approve the scope of the engagement work, including from whom consent and/or community agreement will have to be obtained.

The conviction that the most ethically relevant communities ought to be engaged more robustly and be directly involved in the project’s deliberative processes arise out of the project’s commitment to accountability, a value underwritten by the more fundamental values of respect for persons and justice; both substantive and procedural. Substantive justice entails the sense that there should be an appropriate balancing of the cost and benefits ultimately realised through the research project. Procedural justice prescribes that those who will be subject to the research outcomes are empowered to exert significant influence on the project design and implementation through co-development mechanisms.

Target Malaria's engagement strategy embodies its commitment to the principle of prioritising the most ethically relevant groups, demonstrated for instance by its generous allotment of time and resources to engagement. The role of Target Malaria's international-level engagement with stakeholders and the public has been filled by a single person for a long time. In contrast, around 20 individuals employed by the project are engaging communities in the three African partner countries. An approach focused on political expedience might have committed more resources to the international and pan-African political and regulatory levels, which enjoy more media attention and where more influence can be exerted to expedite the progress of the research. Instead, the project decided to devote greater engagement resources to those groups that are most ethically relevant; the communities which the research is more likely to affect.

Applying this principle has not been without challenges. The communities that face the highest malaria burdens are often rural, sometimes isolated, and in many cases have limited access to formal education and public health facilities. These circumstances pose additional challenges to ensuring that the most relevant communities are equipped and feel empowered to make informed decisions. Most commonly, deliberations related to research and innovation in the field of public health do not include these communities. Others who might have more social or political capital tend to influence those decisions. However, limited access to education and overall social and economic disenfranchisement should not constitute grounds for being excluded from deliberation. The values of accountability, respect for persons, and justice continue to motivate Target Malaria to ensure that affected communities can understand and decide for themselves if, how, and when the project should proceed.

### Conduct engagement in the spirit and form of co-development

Co-development can be defined as "a collaborative process of jointly designing a research pathway and its resultant intervention to reach a common goal" [25]. What makes the relationship between a research project such as Target Malaria's and the communities in which it would operate an ethical imperative? For starters, genuine co-development promotes and results in capacity building in and empowerment of the involved communities.

The NASEM report states:

The ability of people in low-income countries to participate meaningfully in decision making would be supported best not by merely engaging them in decision-making, but by building the capacity in those countries to conduct locally valuable research, regulate and provide oversight of gene drive research generally, and carry out their own decision making about its application. To ensure that capacity-building activities are not just a guise for off-loading expensive and risky research—perpetuating rather than addressing injustice—such activities need to include the development not just of technical capacity to do research but also of capacity to oversee safe and responsible research practices and decide how best to use research findings. Genuine capacity-building must be understood as empowerment, and empowerment must mean that a community or country is able to act on its values rather than merely relying on values imported from elsewhere [23].

This passage aptly describes a significant part of why co-development is both useful and essential. Co-development mitigates against the dynamic of researchers controlling the process and outcome of a project, which is especially important when it involves researchers from high-income countries (HICs) operating in low- and middle-income countries (LMICs). Particularly in relationships involving this kind of power imbalance, building and maintaining the more vulnerable party's trust is important for the ethical legitimacy of the ongoing partnership. Target Malaria's partnerships with local communities in Burkina Faso, Mali, and Uganda reflect this concern.

**Fig. 2:**
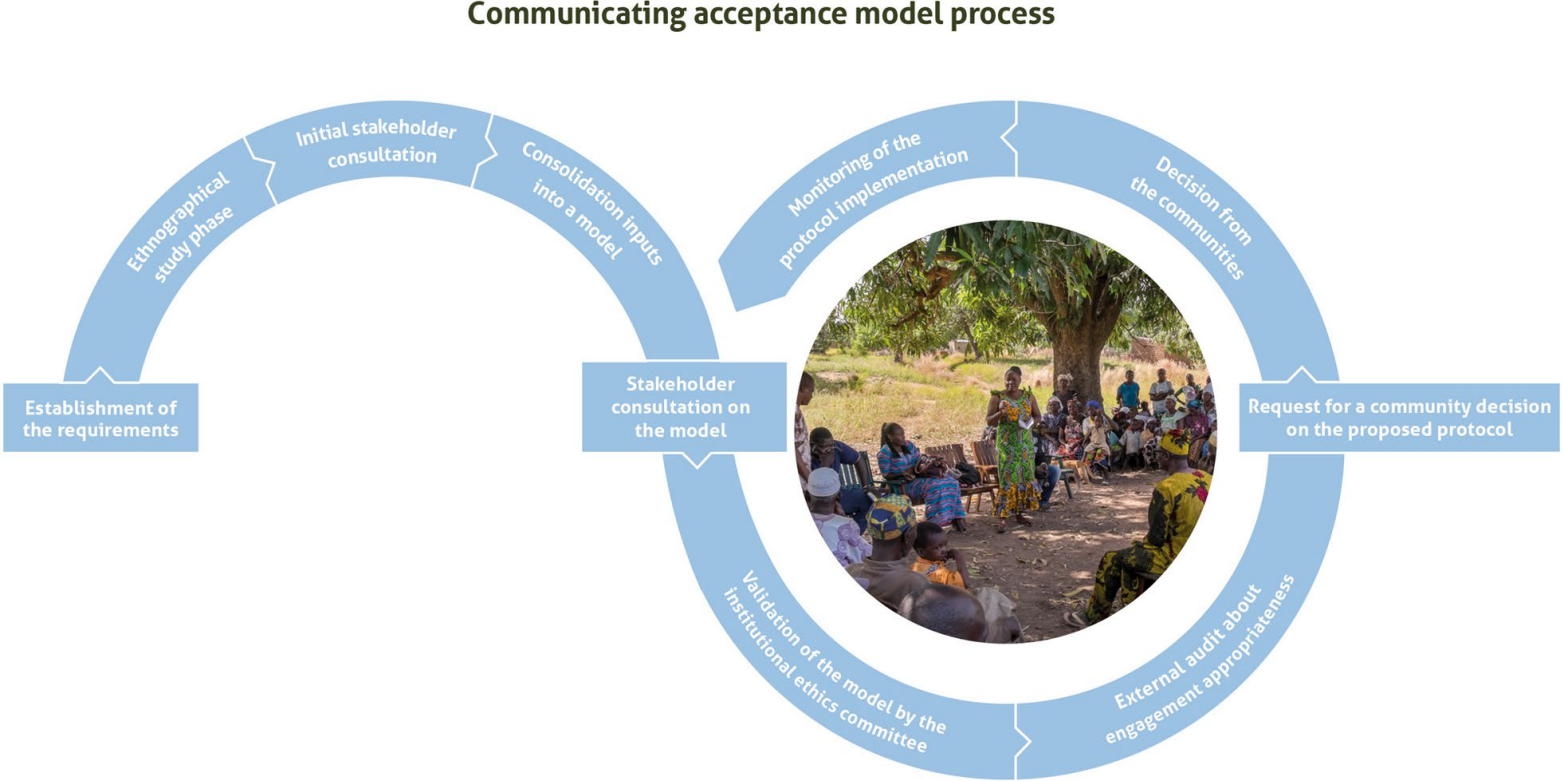
Target Malaria’s co-development process for community agreement

For example, when the project started considering how to achieve meaningful community-level acceptance for some of its activities (initially for mosquito collecting activities in swarms, which occur in the community space), the project chose to co-develop the acceptance model with the community (Fig. 2).

Co-development integrates constraints from both sides. For instance, the research project's need to record the result of the deliberation was accommodated by the community. This process resulted in a model that satisfied both the community and the researchers. It was subsequently approved by an institutional ethics committee, ensuring that the process was co-developed and in line with established obligations of ethical research to protect participant communities. In the project’s guidance documents, engagement with a qualified and appropriate ethics committee is specified as an essential part of a responsible co-development model [13, 14].

In the case of Target Malaria, various ethics committees are involved. At the African partner level, the institutional ethics committee has oversight of overall project activities, which includes reviewing fieldwork protocols as well as engagement protocols, but also ensuring that these protocols are implemented. For example, in the case of the recent release of non-gene drive sterile male mosquitoes in Burkina Faso, the institutional ethics committee observed the release process, thereby providing formal ethical oversight.

In addition, the project has set-up an Ethics Advisory Committee [30, 35]

to provide recommendations on the non-scientific aspects of the research. These include stakeholder engagement activities and a community acceptance model. This committee does not exercise oversight but provides a forum through which the project receives input and criticism about its approach. Its overall objective is learning and improvement.

Co-development looks different from case to case. Ethically designed engagement does not result from a predictable, uniform process. It must be designed on a case-by-case basis and tailored to the communities and research projects which participate in the co-developed engagement process. An ethical engagement process must be a bilateral process. This contrasts with a one-way download of information from the project to the community. The traditional knowledge-deficit engagement model tends towards top-down activities designed to educate the public about the benefits of the technology to secure acceptance or consent for a field evaluation [24]. A co-development relationship should be viewed as essential not only to decision-making processes and a final decision as to *whether* the research will proceed (substantive justice), but also *how* the research should proceed (procedural justice).

For example, deliberative processes vary from culture to culture. What’s ethically mandated in the USA or Europe may not meet local ethical norms in rural Burkina Faso. A given community may not employ democratic methods of deliberation. For various reasons, the methods they rely on may not bestow the same level of voice or agency on all the minority groups that are part of that community. Accepting the results of such deliberative structures may be called into question by an ethics committee or project critics. However, there is no one-size-fits-all ethical discourse on how best to deliberate as a community [36]. Ultimately, to be legitimate, the design of deliberative processes should be sensitive to the context they are designed for [18, 37].

Engaging in co-development can aid the comprehension of processes that are different from one’s own and can serve to bridge practical divides. This, in turn, facilitates a give-and-take process that can accommodate all involved parties by honouring procedural justice in the design of deliberation processes [38]. This way, the process of ethical engagement, honouring the principle of respect for persons, closely mirrors the process of seeking informed consent. It is inappropriate for project researchers or the engagement team to impose deliberative structures on the communities they engage with. It would be inappropriate for a physician conducting clinical research to impose their beliefs about how to deliberate on a clinical research participant. Unless significant internal conflict exists about the deliberative structures in place within the community, challenging these structures from an outsider's position can be seen as disrespectful, potentially creating harmful conflict.

Essentially, co-development is the process by which parties working together towards a common goal practice their partnership through continual open conversation and mutual iteration. Through this process, several essential things may be achieved: the design of mutually agreed upon project processes, increased receptivity and trust on both sides of the partnership, capacity building and empowerment of initially disempowered and vulnerable communities, and ultimately the best research product possible with the least harm or injustice incurred along the way toward achieving it. A positive side effect of co-development done well is that the trust and goodwill fostered between the parties involved can also provide a foundation for enduring friendship and future partnerships.

### Engagement activities should be conducted by representatives of the research project

From Target Malaria’s conclusion that co-development is the most ethical form of engagement for this project, follows that it is internal representatives of the research consortium that should conduct the engagement activities, fulfilling their part of the partnership role as laid out in the section above. As recommended in existing guidance literature, employing a multidisciplinary team which integrates stakeholder engagement, communication, and community mobilization experts is critical [1, 14, 25]. Target Malaria’s engagement work is led by social scientists and engagement practitioners from the countries where the engagement occurs. However, other project scientists also play an important role in engagement. They are frequently trained on how to share their knowledge and listen to stakeholder knowledge in a culturally relevant way [39]. In contrast, some authors have suggested that in order to avoid conflicts of interest, engagement for gene drive research projects should be carried out through 'neutral' third party bodies responsible for convening, facilitating, and recording the outcomes, for instance, during a forum for communities, technology developers, and governments [40]. Convening such a large and disparate group raises serious questions about feasibility. For instance, how to fund the work of such a body, or how to ensure it is and remains neutral? However, such questions will not be explored here. Instead, the paper will look at Target Malaria’s reasons for preferring direct engagement of relevant communities by project representatives.

Target Malaria believes that a project should be co-developed in close partnership with the communities in which it operates and affected by the project’s research activities. Engagement characterized by co-development requires the building of relationships and fostering of trust between the research consortium and the relevant communities, facilitated by direct engagement and partnership activities. Once this trust is earned and maintained, a free flow of information between the partners can begin. Adding what amounts to a 'middleman' to the equation muddles this relationship, adding unnecessary bureaucracy and lengthening communication lines, increasing the opportunity for miscommunication. It also creates a nexus of significant political power in the decision-making process that could be susceptible to influence.

Insisting on the creation of a 'neutral' third party engagement facilitator may sow the seeds of mistrust since the belief that one is required implies that, without such a body, the would-be partners are likely to act in bad faith or are otherwise incapable of interacting together constructively without third party facilitation.

Target Malaria's co-development model is not naïve to potential conflicts of interest, featuring safeguards to mitigate against them. Some of these safeguards come in the form of the ethical principles the project has developed, which include the project’s stated values, the guiding principles mentioned here, in addition to abiding by the required review and approval by an ethics committee each time before proceeding with iterative process changes. The ethics committee provides confidence in the ethical legitimacy of project processes through oversight without getting in the middle of the partnership and complicating communication. As an aside, co-development requires and facilitates engagement of the knowledge of the relevant communities to inform the product and processes of the project. This would be made much more difficult, perhaps impossible if it had to be done through a third-party body. However, specific activities (like evaluations) may require third-party intervention, and likewise, other stakeholders might want to initiate engagement of their own with the communities and/or the researchers.

### Begin engagement early, engage continuously, and iterate often

This principle echoes recommendations found in several articles and guidance documents which address the subject of community engagement for genetically modified mosquito research projects [14, 22, 41, 42]. Target Malaria’s commitment to excellence requires alignment with consensus best practices in the field. “Engaging early and often with regulators” and ensuring that “stakeholders will be engaged at all stages of trials preparation” are some of the core commitments made by researchers for field trials of gene drive organisms [43]. But how early is early enough, or conversely, too early to engage? These questions are often raised in discussions around gene drive, considering that for many projects, funding for engagement is not necessarily available before they have a proof of concept in the laboratory. The challenges of funding and risks associated with early engagement, many years before field evaluation is even envisaged, can deter many researchers. As a result, very often, “engage early” is taken to mean “engage early in the stages *after* you have a working construct and are preparing for a field evaluation”.

When in the project's life cycle should relevant communities be engaged? Co-development requires engagement with the community at a very early stage to avoid the perception that co-development only began once most of the decisions had already been taken. The project’s stepwise approach decided upon in order to empower African researchers, building knowledge through iterative constructs, and building trust and confidence with stakeholders required that Target Malaria’s engagement activities started (in 2014) years before the proof-of-concept gene drive construct was achieved in the lab (in 2019).

Adhering to this principle includes working with vulnerable populations that are the most ethically relevant to the project. This requires a step-by-step engagement process to build the community's understanding of the basic concepts and nature of the science employed by the project so they can make legitimately informed decisions about it [44]. Providing this information required first analysing the communities' existing level of technical knowledge with regards to the proposed genetic approach, including malaria transmission mechanisms, genes, genetic inheritance. This happened before specific information about the mosquito strains that are part of Target Malaria's evaluation pathway was introduced. The process required, as a first step, working towards a mutual understanding with the community about vocabulary and concepts, such as *gene*, *genetic trait*, *genetic inheritance*, and conducting subsequent testing to ensure a mutual understanding of these concepts to a degree that enabled joint decision-making. This was done by co-developing a glossary in local dialects shared by researchers and the community.

As stated above, engagement should facilitate and be characterised by co-development, tailored to and prioritising the most ethically relevant communities. This is a complex and time-consuming process, which must take place in parallel with each successive stage of the project's design and development. Only this way can relevant local community input be effectively harnessed at the earliest designing stage. The engagement processes themselves should also be designed together. For reasons of procedural justice described earlier, this too should happen at the earliest stages of the project’s conception. At a minimum, engagement with relevant communities should occur before bringing any non-engagement-related project activities into the geography of the relevant community [14].

The most fundamental goals of the project should be shaped in partnership with relevant communities prior to the building of significant momentum in any direction. In other words, engagement should begin during the early planning stages of a project's transition from the discovery phase to a development/evaluation phase. This engagement process should pervade all aspects of the project at each stage and inform the fundamental shape and goals of the project itself.

As regards Target Malaria's work in Burkina Faso, Mali and Uganda, communities identified as the most ethically relevant, receiving the most robust engagement, are broadly speaking underprivileged communities with low rates of access to education. Although they have been identified as the most relevant, conducting appropriately thorough, ethically sound engagement with these communities is a resource-intensive process that presents many challenges. Traditionally, engagement has not received adequate budgets. By contrast, between 2016 and 2020, Target Malaria's engagement budget for Burkina Faso represented approximately 23% of the total project budget for that country, as required by the project’s ethical commitments. Reaching this level of funding for engagement activities and for identifying needs and objectives to be fulfilled by subsequent engagement designs was only possible because early-stage fact-finding engagement activities had already been funded and conducted. In short, when it comes to engagement, it’s important to start early. The ethical imperative of making sure this kind of engagement can take place falls mainly on the shoulders of the research funders [45].

A project's ability to engage continuously and iterate often based on outcomes and understandings gained through earlier engagement is contingent on starting early but also on maintaining regular engagement activities. This means that mechanisms should be in place to facilitate regular bi-lateral communication between project personnel and partner communities. This includes complaint response mechanisms. Doing so is of vital ethical importance, as it is through such processes that communities are informed about the project. In turn, the project could become informed and influenced by empowered voices emanating from the community. Only through continuous bi-lateral communication can both partners be updated on the other's needs and opinions to be integrated in decisions made throughout the project life cycle.

In addition to consistent, regular engagement, it is vital that the project be endowed with mechanisms to ensure knowledge engagement [24, 43] and that community opinions discovered through those engagements are iteratively integrated back into the project design and processes. Examples of potential mechanisms for this have been tested by Target Malaria as described by Pare Toe et al. [30] regarding their work in Burkina Faso. If a project fails to develop and enact mechanisms to facilitate this iteration, then for all the information the engagement might produce, they are not effectively performing their role of ensuring a community voice in, and therefore co-development of, the project. It is the authors’ belief that a project which fails in this way also fails in its ethical obligations to respect the people in the communities where the research is conducted. It would represent a failure of procedural justice; a failure to empower and regularly update but also be updated by the communities, crucially denying them a say in how or whether the research project should proceed. Procedural justice and accountability also mean starting engagement early, engaging continuously, and iterating often. It also contributes to substantive justice by avoiding or minimizing harm as well as achieving the greatest possible benefit to communities.

## Conclusion

This paper has aimed to articulate the principles guiding Target Malaria’s engagement strategy, to explain the rationale for selecting these principles, and share some early lessons about their application. Since gene drive technology is an emerging technology with yet to be established best practices in the field, the authors hope by sharing these early lessons to add to the growing literature regarding engagement strategies and practices for area-wide vector control, and more specifically, gene drive research. The principles Target Malaria selected to guide its engagement strategy are:

1. Prioritise engagement with the most ethically relevant groups.
2. Conduct engagement with these groups in the spirit and form of co-development.
3. Engagement should be conducted by representatives of the research project.
4. Begin engagement early, engage continuously, and iterate often.

These principles continue to inform and provide an ethical structure for decisions relating to Target Malaria's engagement strategy and practices.

To date, progress on Target Malaria's testing pathway remains in the early stages of its phased approach, with the goal of environmental release of gene drive modified mosquitoes still years away. Future phases represent novel and hitherto unresolved challenges to designing an ethical engagement strategy. One of these challenges is to appropriately identify, define, and delineate the ethically relevant communities that should be engaged in advance of a mosquito release involving gene drive modification. To address these, a dialogue is required during and after the impact assessment process to ensure a common understanding among stakeholders about who the impacted communities are and their prioritisation in the engagement process. Existing impact assessment best practices put that engagement at the heart of the process [46].

As the geographic area and number of people whose environment will be directly affected by the project expands, the project will face an additional challenge. How can tensions between ensuring culturally appropriate representation in decision-making models and inclusive representation of marginalised groups best be addressed or managed? And what role should external auditors/certifiers play in project activities related to the community acceptance-seeking process and post-release monitoring?

In 2020 the project initiated a consultation process with stakeholder engagement experts, bioethicists, and socio-anthropologists—mainly from Africa—to start an initial conceptualisation of these issues. The principles described here and the values that inform them will be instrumental to devising solutions to these challenging questions. The authors hope that sharing these ethical foundations supporting Target Malaria's engagement strategy will inspire broad reflections about the design of frameworks for ethics-based case-by-case engagement strategies, particularly for area-wide and public health research and applications.

## Data Availability

No data or materials are associated with this article.
